# The Impact of a Ketogenic Diet on Late-Stage Pancreatic Carcinogenesis in Mice: Efficacy and Safety Studies

**DOI:** 10.3390/nu16223919

**Published:** 2024-11-16

**Authors:** Natalia E. Cortez, Tarek A. Bacha, Aya Samir Ead, Cecilia Rodriguez Lanzi, Cassandra Lacroix, Anais Franceschetti, Brian V. Hong, Karen Matsukuma, Gerardo G. Mackenzie

**Affiliations:** 1 Department of Nutrition, University of California, Davis, One Shields Ave, Davis, CA 95616, USAtabacha@ucdavis.edu (T.A.B.); asead@ucdavis.edu (A.S.E.); mcrod@ucdavis.edu (C.R.L.); cassandra.lacroix@agrosupdijon.fr (C.L.); anais.franceschetti@agrosupdijon.fr (A.F.); bvhong@ucdavis.edu (B.V.H.); 2 Department of Clinical and Biological Sciences, 10125 Turin, Italy; 3 Department of Pathology and Laboratory Medicine, University of California Davis School of Medicine, Sacramento, CA 95817, USA; kmatsukuma@ucdavis.edu; 4 Davis Comprehensive Cancer Center, University of California, Sacramento, CA 95817, USA

**Keywords:** pancreatic cancer, ketogenic diet, high-fat diet, pancreatic carcinogenesis

## Abstract

Background: High-fat diets (HFDs) have been associated with an increased risk of pancreatic cancer. In contrast, ketogenic diets (KDs) have been shown to display anti-tumor characteristics. The objective of this work was to evaluate the efficacy of a KD on late-stage pancreatic carcinogenesis in a genetically modified mouse model of pancreatic cancer [LSL-*Kras*^G12D/+^; *Ptf1*-Cre (KC) mice], as well as its liver safety, and to compare it to that of an HFD. Methods: Six-month-old female and male KC mice were randomly allocated to either a control diet (CD) (%kcal: 20% fat, 15% protein, 65% carbohydrates), an HFD (%kcal: 40% fat, 15% protein, 45% carbohydrate) or a KD (%kcal: 84% fat, 15% protein, 1% carbohydrate) and fed these diets for 6 months. Results: HFD-fed, but not KD-fed, mice showed a 15% increase in body weight, plus elevated serum insulin (2.4-fold increase) and leptin (2.9-fold increase) levels, compared to CD-fed mice. At the pancreas level, no differences in pancreatic cancer incidence rates were observed among the diet groups. Regarding the liver safety profile, the HFD-fed mice had higher serum levels of alanine aminotransferase (ALT), aspartate aminotransferase (AST), and alkaline phosphatase (ALP), when compared to the CD and KD groups. In addition, upon histologic examination, an HFD, but not a KD, showed a ~2-fold increase in both macro- and microsteatosis, as well as 35% and 32% higher levels of TLR4 and NF-κB activation, respectively, compared to CD-fed mice. Conclusions: In summary, although a KD intervention alone did not prevent pancreatic carcinogenesis, our data suggests that a KD modulates insulin signaling and hepatic lipid metabolism, highlighting its beneficial effects on healthspan and liver function when compared to an HFD.

## 1. Introduction

Pancreatic ductal adenocarcinoma (PDAC), the most common form of pancreatic cancer, presents a 5-year relative survival of approximately 13% and is projected to become the second leading cause of cancer-related deaths by 2030 [1,2]. The poor overall survival rate is partly due to challenges in early diagnosis, which often results in PDAC patients being diagnosed at an advanced, metastatic stage. Given the limited effectiveness of current treatments, there is a pressing need for better preventive strategies. PDAC can take up to 15–20 years to develop from the occurrence of the first initiating mutational event. Mutational events, such as in the Kras oncogene—present in >90% of PDAC cases—initially give rise to pancreatic intraepithelial neoplasia (PanIN) lesions [3]. Most PDAC cases arise from these microscopic lesions (<5 mm) composed of flat or papillary neoplastic epithelium [4], opening the opportunity for prevention strategies [5]. 

Obesity is a modifiable risk factor that increases the likelihood of developing PDAC by around 20% [6]. In multiple rodent models, intake of high-fat diets (HFDs) can lead to higher insulin levels and inflammation. Furthermore, HFDs have been shown to promote obesity, inflammation, and activation of oncogenic Kras, eventually leading to PDAC [7]. For example, a high-fat, high-calorie diet dramatically increased PanIN formation and PDAC incidence rates in KrasG12D-mutated mice, with a noticeable sex difference, since male mice were more prone to developing high-grade PanIN lesions and PDAC [8]. Similarly, another study demonstrated that an HFD lowered survival in active KrasG12D mice and increased tumor proliferation [9]. Consistent with these findings, we recently observed that an HFD accelerated the early stage of pancreatic carcinogenesis [10].

In contrast to HFDs, ketogenic diets (KDs), characterized by high fat, moderate protein, and very low carbohydrate contents, are used in the treatment of refractory epilepsy [11] and have been studied for their anti-tumor potential [12]. The state of nutritional ketosis, in which the body has adapted to using fats instead of carbohydrates as its main energy source, makes KDs unique compared to other HFDs. The extreme deprivation of carbohydrates in KDs can potentially block cancer cells from using glycolysis, which most tumor cells rely on [13]. Moreover, KDs have been shown to boost survival when combined with radiation [14] and chemotherapy [15]. Hence, a KD could potentially play a beneficial role in cancer prevention. However, a direct comparison between HFDs that do not induce ketosis and those that do (KDs), and the respective enhancing versus preventive roles each could play during pancreatic carcinogenesis, remains unclear. 

In this study, we investigated the impact of a KD in pancreatic cancer development in LSL-KrasG12D/+; Ptf1-Cre (KC) mice, and compared it to that of an HFD with a moderate amount of carbohydrates. Additionally, we explored the effect of these two diets on liver safety profiles in order to assess any potential side effects that could occur as a result of these dietary interventions. While most studies have focused on early pancreatic cancer progression, our research evaluated the direct effects and potential mechanisms by which these diets affect late-stage pancreatic carcinogenesis. 

## 2. Materials and Methods

### 2.1. Animal Study and Dietary Intervention 

All procedures involving animals were approved by the University of California, Davis Animal Care and Use Committee. Genetically engineered female and male KC mice of the C57Bl/6J strain were bred at the UC Davis Animal Facility in Meyer Hall, as previously described [16]. KC mice exhibit ductal lesions similar to human pancreatic intraepithelial neoplasias (PanINs) and progress to metastatic PDAC, closely resembling the pathology observed in human pancreatic cancers [17,18]. 

After weaning, KC mice were individually housed in polycarbonate cages in a room with controlled humidity (40–60%) and temperature (22–24 °C), kept on a 12 h light-dark cycle, and fed chow diet ad libitum LabDiet 5001 (LabDiet, Saint Louis, MO, USA) until 6 months of age, when they were enrolled in the studies. We monitored their body weight monthly until the mice were 12 months old.

At 6 months of age, male and female KC mice (7–14 mice per sex per group) were allocated randomly by the investigators conducting the animal study, in order to maintain a similar baseline body weight among the groups, to either a control diet (CD) (10 males and 10 females; %kcal: 20% fat, 15% protein, 65% carbohydrates], an HFD (7 males and 7 females; %kcal: 40% fat, 15% protein, 45% carbohydrates), or a KD (11 males and 14 females; %kcal: 84% fat, 15% protein, 1% carbohydrate) and were fed one of these diets for 6 months. The exact composition of the diets is shown in Supplemental Table S1. Besides the randomization, other confounders, such as having the same experimenter conduct the same experimental procedure throughout the study, were not controlled. Mice were fed ad libitum, and food was changed three times per week. Body composition was measured using an EchoMRI-100H, provided by EchoMRI LLC (Houston, TX, USA). 

After 6 months of dietary treatment, KC mice were euthanized by carbon dioxide intoxication at a displacement rate of 30% of the chamber’s volume per minute. Serum, as well as the pancreas, liver, lungs, heart, and spleen, were collected, weighed, and stored in liquid nitrogen and 10% buffered formalin. While conducting the methods described below, all investigators were aware of the identity of the samples, except for Dr. Matsukuma, the pathologist on our team.

### 2.2. Metabolic Measurements

Non-fasting glucose levels were measured using a glucometer (Easy Plus II, Home Aid Diagnostics Inc., Deerfield Beach, FL, USA), and ketone body levels (β-hydroxybutyrate) were measured using the Precision Xtra glucose and ketone monitoring system (Abbott, Chicago, IL, USA) following the manufacturer’s instructions.

Blood was rapidly collected via cardiac puncture following euthanasia, and serum was isolated following centrifugation at 3000× *g* for 10 min at room temperature. Total serum cholesterol (Cat. No: 03039773), triglycerides (Cat. No: 20767107 322), alanine aminotransferase (Cat. No: 20764957 322), aspartate aminotransferase (Cat. No: 20764949 322), alkaline phosphatase (Cat. No: 03333752 190), total bilirubin (Cat. No: 05795397 190), albumin (Cat. No:04469658 190), creatinine (Cat. No: 03263991 190), total protein (Cat. No: 03183734 190), and blood urea nitrogen (Cat. No: 04460715 190) were measured using a COBAS INTEGRA kit (Roche, Indianapolis, IN, USA) according to the manufacturer’s instructions.

### 2.3. Histology

After necropsy, pancreas and liver specimens were fixed in 10% buffered formalin overnight at 4 °C. Tissues were processed and embedded by routine methods. Tissue sections (5 μm) were stained with hematoxylin and eosin (H&E) and Masson’s Trichrome (Chromaview, ThermoFisher Scientific, Waltham, MA, USA). Sections were examined using an Olympus BX46 microscope (Olympus America Inc., Center Valley, PA, USA), with 20× and 40× objective lenses by our pathologist (K.M) at the University of California, Davis Comprehensive Cancer Center, in a blinded fashion. Pancreatic tumors were classified by morphologic pattern (glandular, spindled, solid). We also investigated acinar cell loss and used a grading system ranging from 0 to 3 based on the percentage area of acinar cells lost (0: absent, 1: 1–25%, 2: 26–50%, 3: 51–75%, and 4: >75%). Inflammation was based on the average number of lobular inflammatory cells per 40× high-power field (HPF; as counted in 10 non-overlapping HPFs) and graded as 0 = absent, 1 = 1–30 cells, 2 = 31–50 cells, 3 = 51–100 cells, and 4 >100 cells. Fibrosis was quantified based on the cumulative area of stromal fibrosis across the entire pancreas and graded as 0 = absent, 1 = 1–5%, 2 = 6–10%, 3 = 11–20%, and 4 > 20%. 

The liver sections were scored for the presence of macrovesicular and microvesicular steatosis and hepatocyte hypertrophy, according to Liang et al. [19]. Briefly, the severity of macrovesicular and microvesicular steatosis was graded based on the percentage of the total area affected into the following categories: 0 (less than 5%), 1 (5–33%), 2 (34–66%), and 3 (more than 66%). The difference between these two was defined by whether the vacuoles displaced the nucleus to the side (macrovesicular steatosis) or left it centrally positioned (microvesicular steatosis). Similarly, the level of hepatocellular hypertrophy, defined as cellular enlargement more than 1.5 times the normal hepatocyte diameter, was scored, based on the percentage of the total area affected, into the following categories: 0 (less than 5%), 1 (5–33%), 2 (34–66%), and 3 (more than 66%). The evaluation of hepatocellular hypertrophy was based on abnormal enlargement of liver cells, irrespective of rounding of the hepatocytes and/or changes in the cytoplasm or vacuole number, and is, therefore, not a substitute for ballooning. Inflammation was scored based on the number of inflammatory cell clusters (consisting of ≥ 5 lymphocytes) averaged over five fields at 200× magnification, as follows: 0 ≤ 0.5 focus, 1 = 0.5–1.0 focus, 2 = 1.0–2.0 foci, and 3 ≥ 2.0 foci.

### 2.4. Immunohistochemical Staining 

Pancreas and liver tissues were fixed in 10% buffered formalin overnight at 4 °C, then processed and embedded using routine methods. Paraffin sections were deparaffinized, rehydrated, and heated for 12 min at 95 °C in 10 mM (pH 6) citrate buffer (M-15704, ThermoFisher Scientific). Then, tissue sections were incubated with 3% hydrogen peroxide (59105926, Millipore Corporation, Burlington, MA, USA) for 10 min and blocked in animal-free blocker (SP-5030, Vector laboratories, Newark, CA, USA) for 1 h at room temperature, followed by an additional 1 h incubation at room temperature with a primary antibody against PCNA (Cat# 13110, RRID:AB_2636979), F4/80 (Cat #70076, RRID:AB_2799771), p-ERK (Cat# 4370, RRID:AB_2315112), and p-4EBP1 (Cat #2855, RRID:AB_560835). Following that, paraffin sections were incubated with a biotin-conjugated secondary antibody for 30 min at room temperature (856743, Life Technologies, Carlsbad, CA, USA), followed by horseradish peroxidase streptavidin for 30 min at room temperature (856743, Life Technologies), and developed with DAB (SK-4100, Vector laboratories), followed by hematoxylin (MHS16, Sigma, St. Louis, MO, USA) staining. Then, sections were dehydrated, mounted in Cytoseal 60 mounting medium (8310-16, Thermo Scientific), and finally, analyzed using an Olympus BX51 microscope. Scoring involved assessing five or more fields per sample (at magnification ×20), and the percent of positive cells was calculated as previously described [20]. 

### 2.5. Western Blotting

Liver homogenates were prepared, and western blots were performed as previously described [21]. Aliquots of total homogenates containing 25–40 µg protein were separated by reducing 8–12.5% (*w/v*) polyacrylamide gel electrophoresis and electroblotting onto polyvinylidene fluoride membranes. Membranes were blocked in 5% (*w/v*) nonfat milk for 1 h and subsequently incubated with the following antibodies: phospho-protein kinase B (Ser473) (p-Akt) (Cat #4060, RRID: AB_2315049), protein kinase B (AKT) (Cat #9272, RRID: AB_329827), adipose triglyceride lipase (ATGL; Cat #2138, RRID: AB_2167955), phospo-p65 (Ser536) (p-p65) (Cat #3033, RRID:AB_331284), p65 (Cat #8242, RRID:AB_10859369), and toll-like receptor 2 (TLR2) (Cat #12276, RRID: AB_2797867) from Cell Signaling Technologies (Danvers, MA, USA); Sterol regulatory element binding protein 1 (SCREBP1) (sc-13551, RRID: AB_628282), fatty acid synthase (FAS) (sc-74540, RRID: AB_1121387), 3-hydroxymethyl-3-methylglutaryl-CoA synthase HMGCS (sc-373681,RRID: AB_10947237), peroxisome proliferator-activated receptor alpha (PPARα) (sc-398394, RRID: AB_2885073), collagen type 1 alpha 1 chain (Col1A1) (sc-59772, RRID: AB_1121787), and toll-like receptor 4 (TLR4) (sc-293072, RRID: AB_10611320) from Santa Cruz Biotechnology (Santa Cruz, CA, USA); and 3-hydroxymethyl-3-methylglutaryl-CoA lyase (HMGCL) (16898-1-AP, RRID: AB_2295304) from Proteintech Group, Inc. (Rosemont, IL, USA). The membranes were incubated with the antibodies (prepared using a 1:1000 dilution) overnight at 4 °C. After three washing steps with TBS-tween buffer and incubation for 1 h at room temperature in the presence of a secondary antibody (HRP-conjugated), the conjugates were visualized and quantified by chemiluminescence detection using a Chemidoc Imaging-System, Bio-Rad Laboratories (RRID:SCR_008426), Inc. GAPDH (Cat #5174, RRID:AB_10622025) or vinculin (Cat #13901, RRID:AB_2728768) from Cell Signaling Technologies (Danvers, MA, USA), were used as loading controls. The densitometry analysis was performed using the Image J program (RRID:SCR_003070).

### 2.6. Enzyme-Linked Immunosorbent Assay (ELISA)

ELISA for murine insulin in serum was performed following the manufacturer’s protocols (Crystal Chem, Inc., Elk Grove Village, IL, USA; Cat #90080). Briefly, diluted serum samples (1/5) were added to an insulin-coated 96-well plate. Following 2.5 h incubation, the wells were washed, and a biotinylated anti-mouse insulin antibody was added and incubated for an additional hour. After three washes, an HRP-conjugated streptavidin was added. After 30 min of incubation, wells were washed three times, TMB substrate solution was added to the wells, and color developed in proportion to the amount of insulin bound.

### 2.7. Statistical Analysis

The data were expressed as mean ± SD. Data were checked for normality and homogeneity before conducting the statistical analysis test. Statistical evaluation was performed by one-factor analysis of variance (ANOVA) or two-factor ANOVA followed by the Tukey test adjusted for multiple comparisons. A *p*-value of < 0.05 was regarded as statistically significant. Data analyses were performed by GraphPad (Prism version 9.2, RRID:SCR_002798).

## 3. Results

### 3.1. Effect of a High-Fat Diet and a Ketogenic Diet on Metabolic Parameters in KC Mice

**Figure 1:**
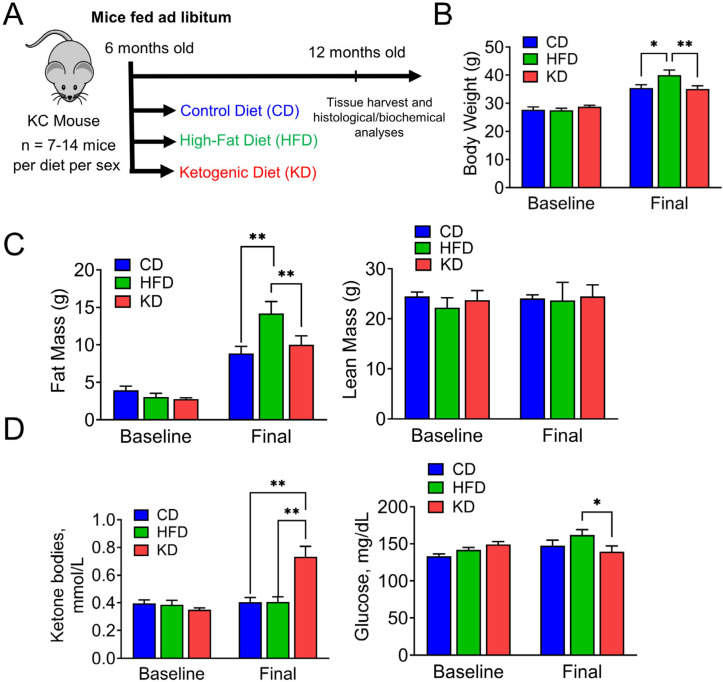
Effect of an HFD and KD on body weight, body composition, and ketone body levels in KC mice. (**A**) Scheme of the study design. (**B**) Baseline and final body weight. (**C**) Fat and lean mass of KC mice fed a control diet (CD), high-fat diet (HFD), or ketogenic diet (KD) are shown. (**D**) β-hydroxybutyrate and glucose levels at the baseline and endpoint are presented. Values represent the mean with ± SD (n = 12–23 animals/group). * *p* < 0.05 and ** *p* < 0.01 were calculated using two-way ANOVA. Baseline and final levels were measured when KC mice were 6 and 12 months old, respectively.

To investigate the effects of the dietary interventions on late-stage pancreatic carcinogenesis, cohorts of male and female 6-month-old KC mice were randomly assigned to one of three diets: a control diet (CD), a high-fat diet (HFD), or a ketogenic diet (KD), and fed these diets ad libitum for an additional 6 months (Figure 1A). At the endpoint, the HFD-fed mice had a significant increase in body weight (mean ± SD) compared to both CD- and KD-fed mice (40.0 ± 6.9 g vs. 35.4 ± 5.1 g; *p* < 0.05 and 40.0 ± 6.9 g vs. 35.1 ± 5.6 g, *p* < 0.01, respectively; Figure 1B). The increased body weight was accompanied by a statistically significant higher fat mass weight (g) in HFD-fed mice (mean ± SD), compared to both CD- and KD-fed mice (14.2 ± 5.5 vs. 8.8 ± 4.1 and 14.2 vs. 10.0 ± 5.7, respectively; *p* < 0.01 for both; Figure 1C). No significant changes were observed in lean mass among the groups (Figure 1C).

To validate the effectiveness of the KD, we measured blood ketone body levels, specifically assessing β-hydroxybutyrate (ΒHB) levels. As expected, while baseline levels of ΒHB were comparable among the three diet groups, we observed significantly elevated levels of ΒHB (mmol/L) in KD-fed mice (mean ± SD), compared to both CD- and HFD-fed mice (0.73 ± 0.37 vs. 0.40 ± 0.15 and 0.73 ± vs. 0.41 ± 0.15, respectively; *p* < 0.01 for both; Figure 1D). Additionally, non-fasting blood glucose levels (mg/dL) were significantly reduced in KD-fed mice compared to HFD-fed mice (139 ± 26 vs. 162 ± 35, *p* < 0.05; Figure 1D). 

### 3.2. A High-Fat Diet Significantly Increased Pancreas and Liver Weight Compared to a KD

**Figure 2:**
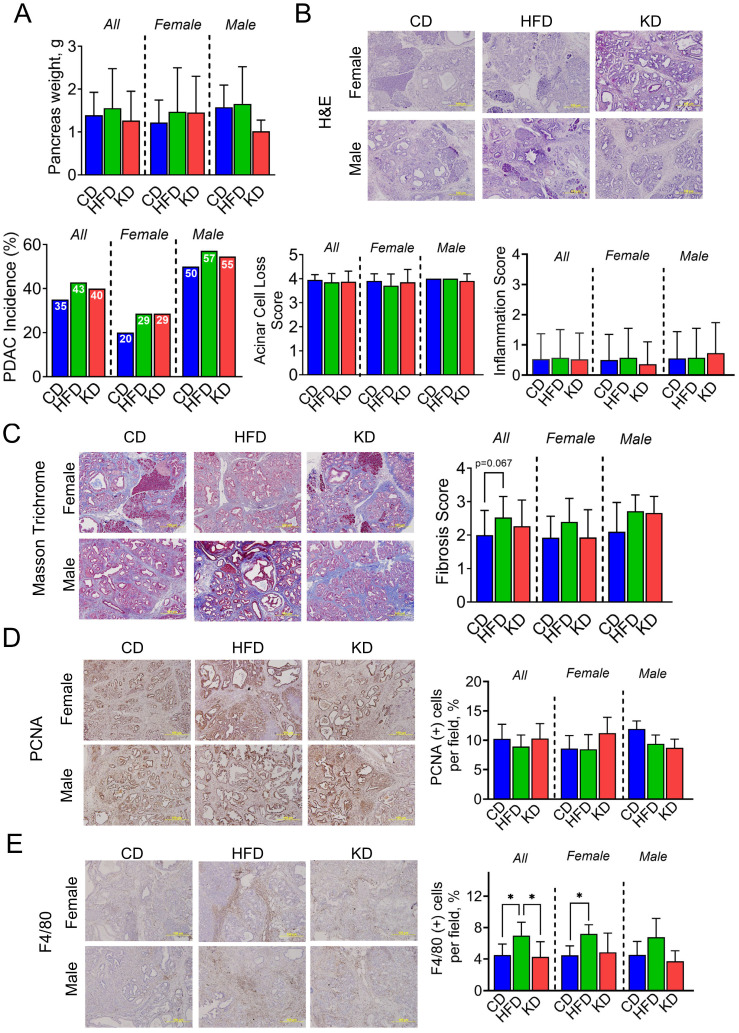
Effect of a KD and HFD on PDAC incidence rates in mice. (**A**) Pancreas weight of KC mice fed a control diet (CD), high-fat diet (HFD), or ketogenic diet (KD). (**B**) Pancreas histology using H&E-stained tissues of KC mice fed the control diet (CD), high-fat diet (HFD), or ketogenic diet (KD) in 12-month-old female and male KC mice. For each diet, images (×20) from female and male mice were shown. In addition, cancer incidence (%), acinar cell loss, and inflammation scores are shown. (**C**) Pancreas histology using Masson Trichrome-stained tissues of KC mice fed the control diet (CD), high-fat diet (HFD), or ketogenic diet (KD) in 12-month-old female and male KC mice. For each diet, images (×20) from female and male mice livers are shown, and fibrosis scores were quantified. (**D**) IHC for PCNA was performed on KC pancreas tissue sections, and photographs were taken at 20× magnification. Representative images are shown. (**E**) IHC for F4/80 was performed on KC pancreas tissue sections, and photographs were taken at 20× magnification. Representative images are shown. Results were expressed as a percentage of PCNA+ cells ± SD per × 20 fields (n = 7–14 animals/group/sex). * *p* < 0.05, data are depicted as mean with ± SD, one-way ANOVA.

After the 6-month dietary intervention, we assessed the effect of the diets on organ weights. Male mice fed an HFD had higher pancreas weights than KD-fed males (1.70 ± 0.80 g vs. 1.01 ± 0.26 g; *p* < 0.05) (Figure 2A). In addition, liver weight was significantly higher (mean ± SD) in HFD-fed mice than in KD-fed mice (1.71 ± 0.52 g vs. 1.46 ± 0.33 g; *p* < 0.05), and this trend was also observed specifically in male mice (2.10 ± 0.45 g vs. 1.52 ± 0.43 g; *p* < 0.05) (Supplemental Figure S1A). On the other hand, HFD- and KD-fed mice displayed significantly increased lung weights compared to the CD group, and these differences were observed specifically in female mice (*p* < 0.05) (Supplemental Figure S1B). Finally, no significant differences were observed in heart and spleen weight across the three diet groups (Supplemental Figure S1C,D).

### 3.3. Effect of a High-Fat Diet and a Ketogenic Diet on PDAC Incidence Rates in KC Mice

Histological techniques were performed to quantify PDAC incidence rates in KC mice fed the respective diets for six months. The PDAC incidence rates were similar across all diet groups at 12 months of age, with rates of 35%, 43%, and 40% in CD-, HFD-, and KD-fed mice, respectively (Figure 2B). Interestingly, male KC mice across all diet groups experienced double the PDAC incidence rates compared to female KC mice. For KC females, the PDAC incidence rate was 20%, 29%, and 29% in CD-, HFD-, and KD-fed mice, respectively, while for males, it was 50%, 57%, and 55% (Figure 2B). We then evaluated the effect of dietary intervention on acinar cell loss, inflammation, and fibrosis. Mice across all diets experienced significant acinar cell loss and similar degrees of inflammation (Figure 2B). To determine if there was an effect on fibrosis, we quantified the levels of collagen deposition in the pancreas using Masson Trichrome staining (Figure 2C). Although we observed no significant differences in fibrotic scores among the groups, there was an increasing trend in HFD-fed KC mice, compared to the CD and KD groups (Figure 2C). Consistent with the similar levels of PDAC incidence and acinar-to-ductal metaplasia, the levels of proliferating cell nuclear antigen (PCNA) staining were similar among the three groups (Figure 2D). When separated by sex, PCNA staining was still comparable in males and females (Figure 2D).

We then assessed the effect of a KD and an HFD on macrophage infiltration. We observed that HFD-fed mice displayed higher macrophage infiltration compared to CD- and KD-fed mice (Figure 2E).

### 3.4. A High-Fat Diet Significantly Increased Serum Insulin and Leptin Levels Compared to a KD

**Figure 3:**
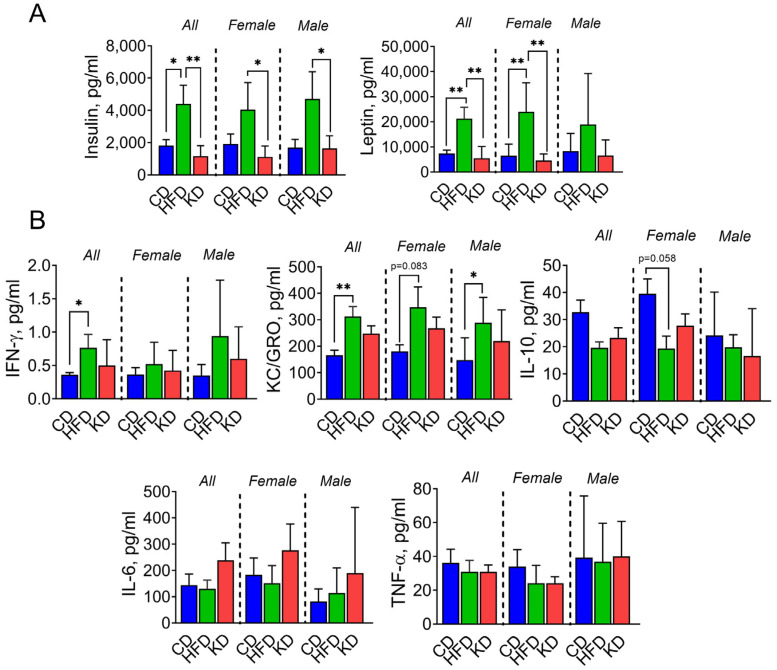
An HFD significantly increased serum insulin and leptin levels compared to a KD. (**A**) Serum insulin and leptin levels after 6 months in their respective diets. (**B**) Cytokine and chemokine levels were measured in serum in 12-month-old KC mice fed either a control diet (CD), a high-fat diet (HFD), or a ketogenic diet (KD). * *p* < 0.05, and ** *p* < 0.01, data are depicted as mean with ± SD, one-way ANOVA (n = 5–12 animals/group/sex).

We assessed the impact of dietary intervention on hormonal and cytokine levels in KC mice by measuring insulin and leptin levels, as well as levels of IFN-γ, KC/GRO, IL-10, IL-6, and TNF-α. HFD-fed mice showed significantly elevated insulin and leptin levels compared to both CD and KD groups, and insulin levels were significantly higher in HFD-fed males than females in KD-fed mice. Leptin levels were significantly higher in HFD-fed groups when compared with CD and KD groups, and leptin levels were higher in HFD-fed females than males (Figure 3A). HFD-fed mice also displayed elevated IFN-γ and KC/GRO levels compared to CD-fed mice. In contrast, no significant differences in IFN-γ and KC/GRO levels were observed between HFD- and KD-fed mice. Moreover, no significant differences in IL-10, IL-6, and TNF-α levels were observed among the three diet groups (Figure 3B). 

**Figure 4:**
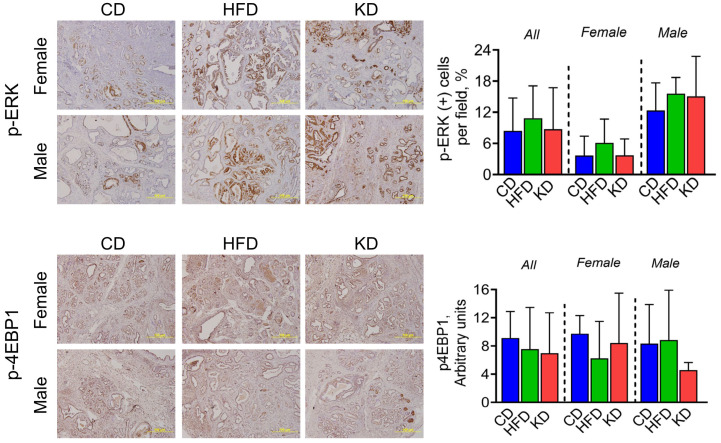
Effect of an HFD and KD on pathways downstream of insulin signaling. IHC for pERK and p4EBP1 was performed on KC pancreatic tissue sections, and photographs were taken at 20× magnification. Representative images are shown. Results were expressed as a percentage of pERK+ and p4EBP1+ cells ± SD per ×20 fields. Data are depicted as mean with ± SD, one-way ANOVA (n = 3–6 animals/group/sex).

Insulin signaling has been implicated in driving cell proliferation and has been shown to be upregulated in PDAC [22]. Therefore, we assessed the RAS/RAF/ERK and PI3K/AKT/mTOR pathways by immunohistochemistry, as they are downstream of insulin signaling. We observed no significant changes in ERK and 4EBP1 phosphorylation levels among the three diets (Figure 4). 

### 3.5. A High-Fat Diet, but Not a Ketogenic Diet, Affects Liver Function in KC Mice

**Figure 5:**
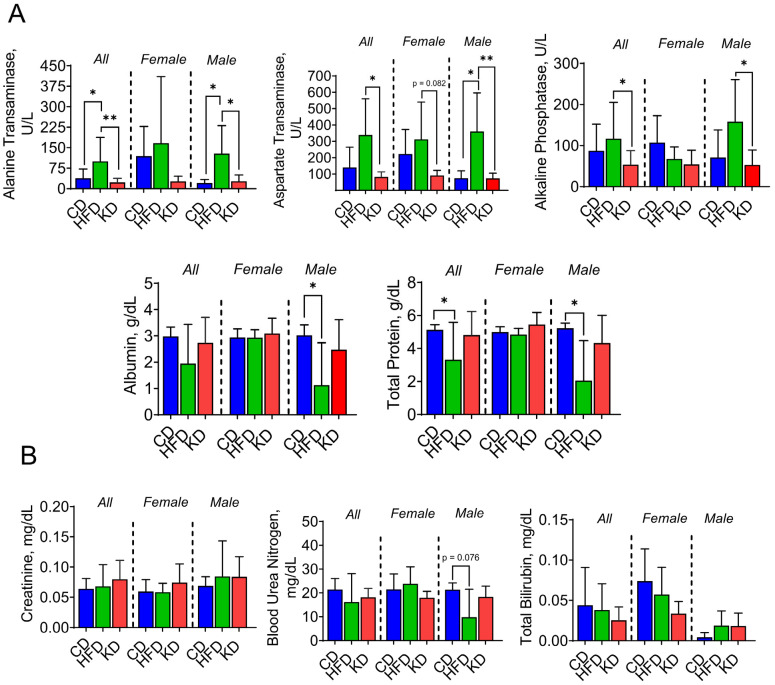
An HFD, but not a KD, affects liver function in KC mice. (**A**) Final liver enzymes, protein, and (**B**) waste product levels were measured in 12-month-old KC mice fed either a control diet (CD), a high-fat diet (HFD), or a ketogenic diet (KD). * *p* < 0.05, and ** *p* < 0.01, data are depicted as mean with ± SD, one-way ANOVA (n = 3–7 animals/group/sex).

To determine whether a KD or an HFD affected liver function, we measured endpoint serum levels of the liver enzymes alanine transaminase (ALT), aspartate aminotransferase (AST), and alkaline phosphatase (ALP). Albumin and total protein levels were measured as markers of liver protein metabolism. HFD intake led to significantly increased levels of ALT, ASP, and ALP, when compared to CD and KD groups (Figure 5A). Furthermore, HFD-fed KC mice experienced reduced albumin and protein levels compared to CD-fed mice, which was driven by the effect observed in males (Figure 5A). We also assessed kidney function by measuring bilirubin, blood urea nitrogen, and creatinine levels, yet no significant changes were observed among the three diet groups (Figure 5B).

### 3.6. A Ketogenic Diet Prevents Liver Lipids Accumulation in Male KC Mice

**Figure 6:**
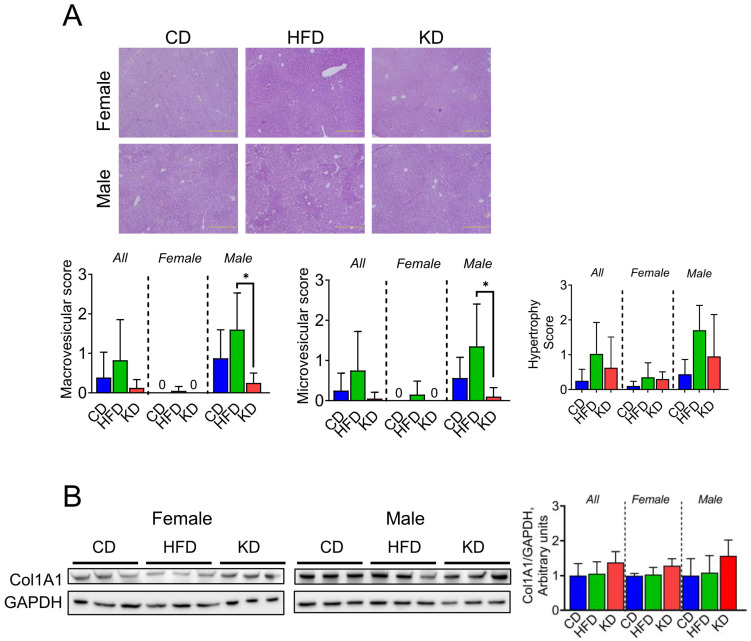
Effect of a KD on liver lipids accumulation. (**A**) Liver histology using H&E-stained tissues of KC mice fed the control diet (CD), high-fat diet (HFD), or ketogenic diet (KD) in 12-month-old female and male KC mice. For each diet, images (×20) from female and male mice were shown. Macrovesicular, microvesicular, and hypertrophy scores are shown. (**B**) Protein levels of fibrotic marker Col1A1 analyzed by western blotting in liver lysates from 12-month-old KC mice fed a CD, HFD, or KD. GADPH was used as a loading control. Each lane represents an individual mouse, and three mice per group are shown. Bands were quantified, and results are expressed as percentage control. * *p* < 0.05, data are depicted as mean with ± SD, one-way ANOVA (n = 4–6 animals/group/sex).

Given that excessive fat intake has the potential to trigger hepatocellular injury, resulting in enzyme induction and the development of macrovesicular and microvesicular steatosis as well as hypertrophy/ballooning, we determined the impact of the three dietary interventions on hepatic lipid accumulation and steatosis. For this purpose, we performed liver staining with hematoxylin and eosin and evaluated histological changes in CD, HFD, and KD groups, specifically examining patterns of hepatocyte injury (Figure 6A). Using a scoring system established by Liang et al. [19], we assessed the presence of macrovesicular and microvesicular steatosis, as well as hepatocyte hypertrophy. We observed a significant increase in macrovesicular and microvesicular steatosis in male KC mice fed an HFD compared to those fed a KD (Figure 6A). Of note, no differences in steatosis and hepatocyte hypertrophy were observed in female mice (Figure 6A). We also compared the levels of collagen type I alpha 1 (COL1A1) protein, a biomarker for liver fibrosis, and no significant differences were observed among the three groups (Figure 6B). 

### 3.7. Effects of a High-Fat Diet and a Ketogenic Diet on Ketogenesis and on De Novo Lipogenesis in the Liver

**Figure 7:**
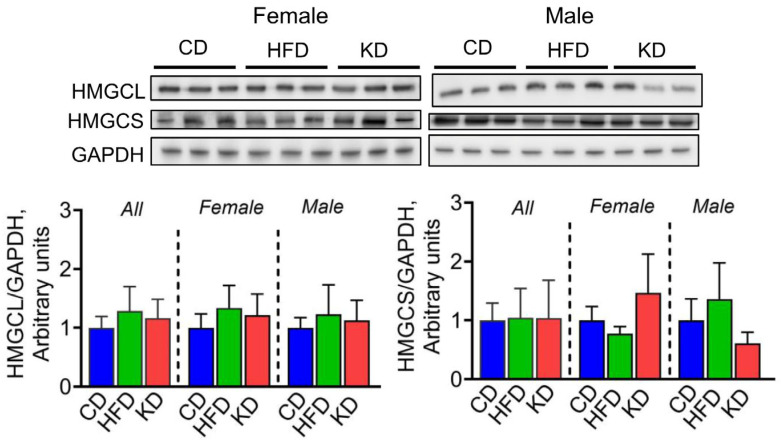
Effect of a KD on ketogenesis and glucose metabolism. Protein levels of HMGCL and HMGCS analyzed by western blotting in liver lysates from 12-month-old KC mice fed the control diet (CD), high-fat diet (HFD), or ketogenic diet (KD). Image shows representative immunoblots of HMGCL and HMGCS and protein levels of GADPH used as a loading control. Each lane represents an individual mouse, and three mice per group are shown. Bands were quantified, and results are expressed as percentage control. Data are depicted as mean with ± SD, one-way ANOVA (n = 4–6 animals/group/sex).

We then investigated the regulation of enzymes involved in ketogenesis following dietary intervention. No significant differences in hepatic levels of HMGCL nor HMGCS were observed among the three groups (Figure 7).

**Figure 8:**
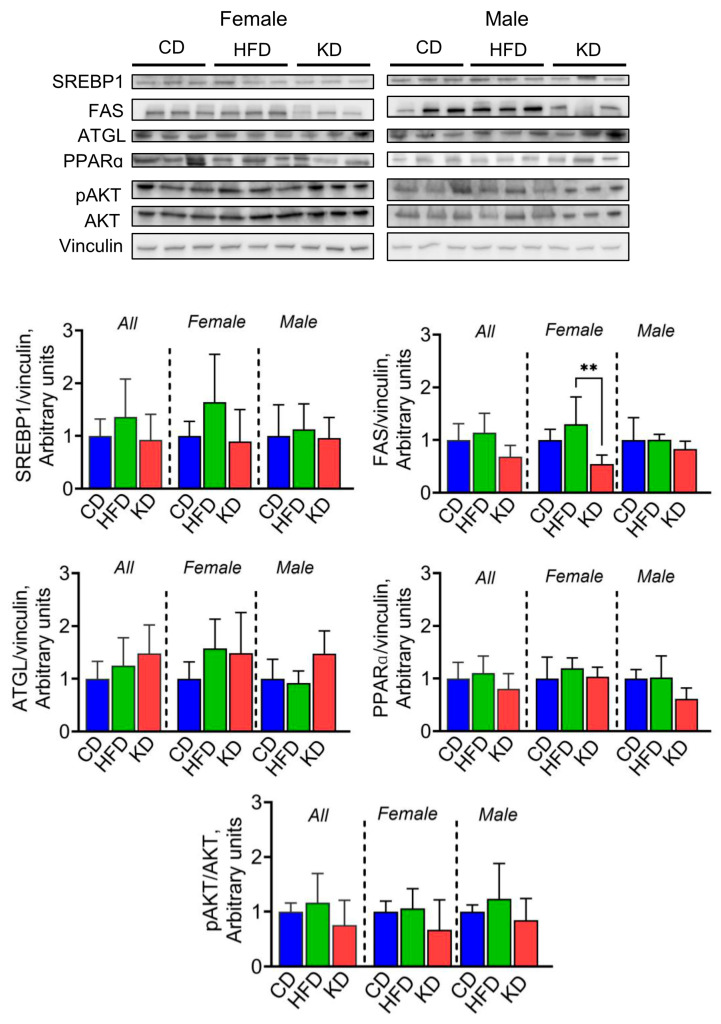
Effect of an HFD and KD on de novo lipogenesis. Protein levels of SREBP1, FAS, ATGL, PPARα, and pAKT analyzed by western blotting in liver lysates from 12-month-old KC mice fed the control diet (CD), high-fat diet (HFD), or ketogenic diet (KD). Image shows representative immunoblots of SREBP1, FAS, ATGL, PPARα, and pAKT and protein levels of vinculin used as a loading control. Each lane represents an individual mouse, and three mice per group are shown. Bands were quantified, and results are expressed as percentage control. ** *p* < 0.01, data are depicted as mean with ± SD, one-way ANOVA (n = 3–6 animals/group/sex).

Then, we explored whether a KD could influence the protein levels of sterol regulatory element-binding proteins (SREBPs), which, together with the FAS enzyme, are key regulators of de novo lipogenesis [23,24]. There were no significant differences in SREBP1 protein levels among groups. However, female mice fed a KD showed significantly lower FAS protein levels compared to those treated with an HFD (Figure 8). In addition, no significant differences in ATGL levels were observed among the three diet groups. We then assessed the levels of PPARα, a key nuclear receptor regulating de novo lipogenesis [25]. No significant differences were observed when compared to HFD-fed mice (Figure 8). Finally, we assessed AKT phosphorylation levels, given AKT’s role in stimulating lipid synthesis via SREBP activation [26]. No significant differences in AKT phosphorylation levels were observed among the three diet groups (Figure 8).

### 3.8. A High-Fat Diet, but Not a Ketogenic Diet, Increases TLR4 and NF-κB Activation in the Liver

**Figure 9:**
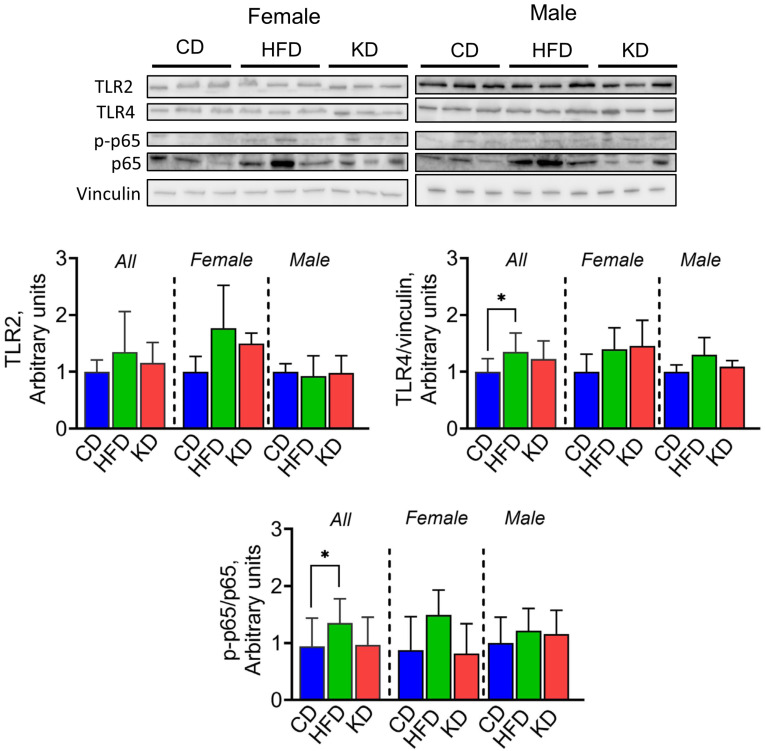
Effect of a KD on markers of inflammation in the liver of KC mice. Protein levels of TLR2 and TLR4, and p-p65 analyzed by western blotting in liver lysates from 12-month-old KC mice fed a control diet (CD), high-fat diet (HFD), or ketogenic diet (KD). Image shows representative immunoblots of TLR2 and TLR4, and p-p65 and protein levels of vinculin used as a loading control. Each lane represents an individual mouse, and three mice per group are shown. Bands were quantified, and results are expressed as percentage control. * *p* < 0.05, data are depicted as mean with ± SD, one-way ANOVA (n = 4–6 animals/group/sex).

Finally, we evaluated the effect of a KD and an HFD on inflammatory markers in the liver. The activation of Toll-like receptor (TLR) signaling is key in the process of liver inflammation and fibrosis, with TLR4 and TLR2 serving crucial roles in the progression of non-alcoholic steatohepatitis [27]. Therefore, we measured, by western blotting, if an HFD or a KD could affect the activation of TLR2 and TLR4, and its downstream NF-κB activation. While TLR2 protein levels did not differ significantly among the groups, the HFD-fed group showed significantly higher TLR4 and p65 phosphorylation levels compared to the CD group when all mice were analyzed together (Figure 9). 

## 4. Discussion

Substantial evidence supports the notion that an HFD intake increases the risk of developing cancer, including PDAC [28]. However, there is a lack of understanding regarding the potential use of a KD as a beneficial dietary intervention for PDAC and its underlying mechanisms. In this study, we evaluated the impact of a KD on late-stage pancreatic carcinogenesis and compared it to that of an HFD with a moderate amount of carbohydrates.

An unexpected finding from our study was that PDAC incidence rates were similar in KC mice fed either a CD, HFD, or KD for 6 months. Prior preclinical studies have consistently shown increased PDAC incidence rates upon feeding an HFD. However, these studies have introduced the dietary interventions when mice were young (around 6 weeks of age) [10,28], compared to our late dietary intervention (when mice were 6 months of age). It is noteworthy that KC mice fed a chow diet develop PDAC at around 1 year of age [28]. It might be the case that by the time dietary interventions were introduced, KC mice were already at a carcinogenic stage where diet alone had no major impact on the ultimate development of PDAC.

An unexpected finding was the increase in lung weight in the KC mice fed the HFD or KD. Given that the lungs are a primary site for PDAC metastasis, the increase in lung weight could have suggested metastasis to this organ. Although diets rich in fat have been shown to increase PDAC growth and metastasis [29], we did not observe any lung metastasis in any dietary group. Further research is needed to elucidate the reason for the higher lung weight after diets rich in fat.

Despite the lack of impact in decreasing pancreatic cancer incidence, a KD intake showed multiple beneficial effects. For example, KD-fed mice had significantly lower body weight, insulin, and leptin levels compared to HFD-fed mice. Increased insulin and leptin levels have been previously observed in diet-induced obese mice with pancreatic cancer [30,31]. In our model, unlike with an HFD, a KD did not promote weight gain, hyperleptinemia, or insulin resistance. These findings are supported by a study of non-alcoholic fatty liver disease patients who experienced a 53% reduction in serum insulin and 45% in plasma leptin levels with a KD [32]. Thus, in contrast to an HFD, implementing a KD does not increase the risk of insulin resistance.

Elevated levels of cytokines such as IFN-gamma, IL-6, IL-10, and TNF-alpha have been associated with an increased risk of pancreatic cancer [33,34]. This association is attributed to the tumor-promoting inflammation and tumor-suppressive immunity characteristic of the pancreatic tumor microenvironment [35]. In our study, we observed a significant increase in IFN-gamma in HFD-fed mice, but no changes in IL-6, IL-10, or TNF-alpha levels across the diets. Similarly, another study observed that in comparison to a CD, an HFD led to a positive enrichment of gene sets related to IFN-γ response [36]. Additionally, we observed an increase in KC/GRO secretion in HFD-fed KC mice. These chemokines have been shown to promote the progression and metastasis of pancreatic cancer [37], and elevated levels of these chemokines have been associated with poor prognosis in pancreatic cancer patients. Although we did not observe a direct impact on pancreatic cancer incidence among the dietary groups, our findings are aligned with previous research that shows that HFDs exacerbate inflammatory responses and metabolic disturbances [31].

Concerns have been raised about the potential long-term side effects of KD, particularly regarding liver toxicity [38]. However, recent evidence suggests otherwise, demonstrating reduced liver steatosis, oxidative stress, inflammation, and fibrosis following intake of a KD [39,40,41]. Hence, we assessed multiple parameters of liver toxicity in the mice fed an HFD and a KD. While feeding an HFD increased liver enzyme activity in KC mice, there were no changes in liver enzymes nor in markers of kidney function with the KD. Consistent with our findings, liver and kidney function tests remained within the normal range in rats fed a KD for 2 months [42]. 

Increased risk of hepatic steatosis is another potential long-term concern of feeding a KD that we addressed in our study [43]. Instead, our results showed that male KD-fed mice experienced reduced steatosis in contrast to those fed an HFD, which is consistent with previous findings [39]. Additionally, our results have shown that a KD impacts de novo lipogenesis by decreasing FAS expression in female mice in comparison to an HFD. Such results in FAS expression are consistent with prior studies that suggest that decreased insulin signaling can lead to decreased lipogenesis [44].

The activation of TLR signaling plays a key role in hepatic inflammation and fibrosis, with TLR4 and TLR2 having essential roles in the progression of non-alcoholic steatohepatitis [27]. In contrast to an HFD, the KD presented anti-inflammatory properties, showing lower levels of TLR4 and p65 phosphorylation. This is consistent with other studies that have shown the anti-inflammatory properties of a KD, as well as protective effects against nonalcoholic fatty liver disease [45,46,47,48,49]. Therefore, our findings highlight a clear distinction of the detrimental effect of HFDs, but not KDs, on liver health. As research progresses, these findings may lead to more effective dietary prevention and/or treatment strategies for fatty liver disease in humans.

This study has some limitations, such as the inability to diagnose the stage of carcinogenesis prior to intervention, the evaluation of cancer incidence at only one late timepoint (when the majority of KC mice might have already developed cancer), and the small sample size due to the complexity of using genetically engineered mouse models. Finally, we cannot dismiss the possibility that many of the observed differences in the assessed parameters between males and females may stem from insufficient power to detect sex-dependent effects. Even though our power calculations were informed by previous studies feeding an HFD to KC mice [28], it is possible that starting the study at a later stage affected the primary outcomes.

## 5. Conclusions

Although feeding a KD did not directly mitigate pancreatic carcinogenesis, our data indicate that a KD positively modulates insulin signaling and the hepatic metabolism of lipids in late-stage pancreatic carcinogenesis. In addition, a KD showed a favorable hepatic safety profile when compared to an HFD. These findings suggest the importance of comparative dietary studies to better understand the impact of such interventions on the healthspan of individuals with cancer risk. Moreover, our data highlights the potential benefit of evaluating the use of KDs compared to HFDs in clinical settings. Future studies should investigate the sex-specific effects of KDs, and explore whether KDs with varying fatty acid compositions or interventions at earlier times could serve as a potential strategy to prevent pancreatic carcinogenesis.

## Data Availability

The data used and analyzed in the current study are available from the corresponding author upon request. The data are not publicly available due to technical and time limitations.

## Supplementary Materials

The following supporting information can be downloaded at: https://www.mdpi.com/article/10.3390/nu16223919/s1, Figure S1: Organ weights for liver, lungs, heart, and spleen; Table S1: Composition of all three diets.
