# Supplementary material for: The Impact of a Ketogenic Diet on Late-Stage Pancreatic Carcinogenesis in Mice: Efficacy and Safety Studies
Source: Nutrients. 2024 Nov 16;16(22):3919. doi: 10.3390/nu16223919 (PMC11597385; doi:10.3390/nu16223919)
Supplement: Supplementary file 1 [file nutrients-16-03919-s001.zip › nutrients-3300946-supplementary.pdf]

Supplemental Table S1. Composition of all three diets.

CONTROL	Ingredients	g/kg	kcal/kg	%kcal	
**Fat**	soybean oil	87	748.2	19.80%	**20%**
	fat in casein	*0.972*	*8.748*	0.20%	
**Carbohydrate**	corn starch	400	1520	40.20%	**65%**
	maltodextrin	130	494	13.10%	
	sucrose	100	380	10.00%	
	mineral mix TD94046	35	29.3	0.80%	
	vitamin mix CA40060	20	35.5	0.90%	
**Protein**	casein	162	554.04	14.60%	**15%**
	DL-methionine	2.6	12.74	0.30%	
	cellulose	64	0	0.00%	
	TBHQ	0.013	0	0.00%	
	Total	*1001.6*	*3782.5*	*100.00%*	

KETOGENIC	Ingredients	g/kg	kcal/kg	%kcal	
**Fat**	soybean oil	90	774	12.50%	**84%**
	lard	480	4416	71.30%	
	fat in casein	*1.6*	*14.5*	*0.20%*	
**Carbohydrate**	vitamin mix CA40060	20	35	0.60%	**1%**
	corn starch	7.5	28.5	0.50%	
**Protein**	casein	268	916.6	14.80%	**15%**
	DL-methionine	2.6	12.74	0.20%	
	calcium phosphate dibasic	19	0	0.00%	
	calcium carbonate	8	0	0.00%	
	cellulose	89	0	0.00%	
	TBHQ	0.13	0	0.00%	
	mineral mix TD79055	20	0	0.00%	
	Total	996.7	6197.8	100.00%	

HIGH FAT	Ingredients	g/kg	kcal/kg	%kcal	
**Fat**	soybean oil	70	602	12.70%	**40%**
	lard	140	1288	27.10%	
	fat in casein	*1.23*	*11.07*	0.20%	
**Carbohydrate**	corn starch	350	1330	28.00%	**45%**
	maltodextrin	100	380	8.00%	
	sucrose	100	380	8.00%	
	mineral mix TD94046	20	16.7	0.40%	
	vitamin mix CA40060	20	35.5	0.70%	
**Protein**	casein	205	701.1	14.70%	**15%**
	DL-methionine	2.6	12.74	0.30%	
	cellulose	50	0	0.00%	
	TBHQ	0.13	0	0.00%	
	Total	*1007.6*	*4757.1*	100.00%	

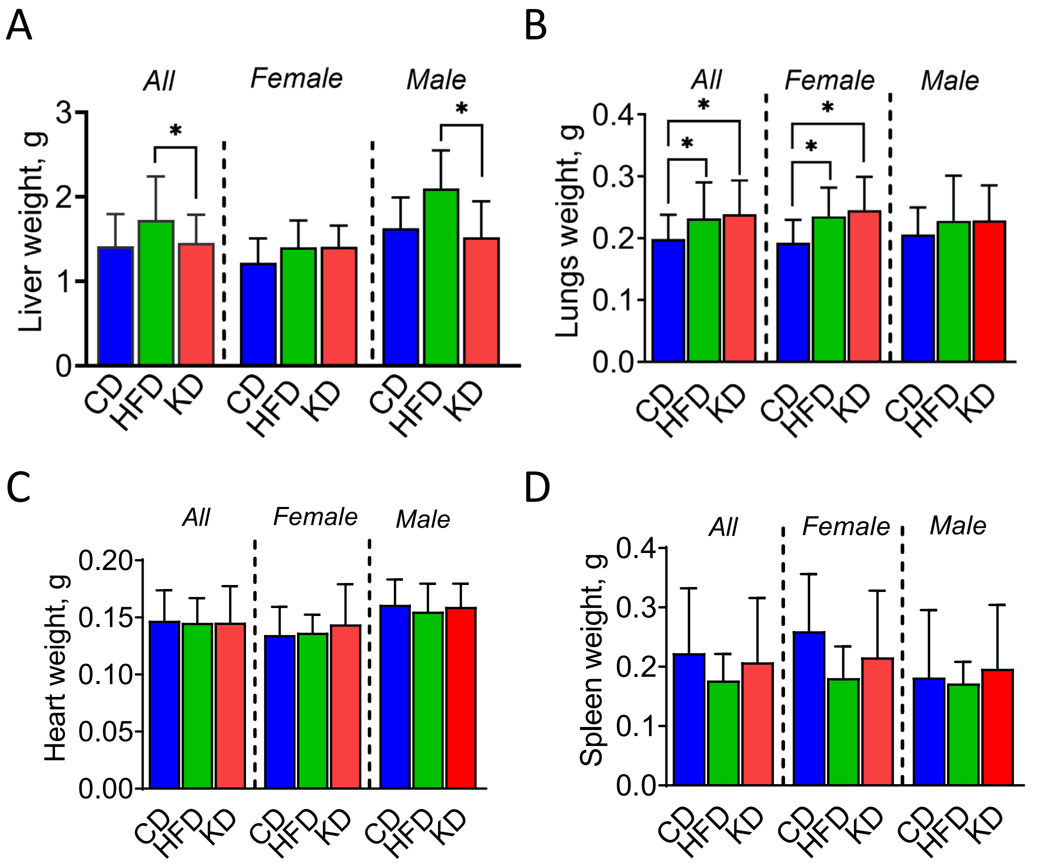

Supplemental figure S1: Organ weights for **A-** liver, **B-** lungs, **C-** heart, and **D-** spleen. Values represent the mean with ± SD. *P <0.05; one-way ANOVA (n= 7-14 animals/group/sex).
